# Various Strategies for Pain-Free Root Canal Treatment

**Published:** 2013-12-24

**Authors:** Masoud Parirokh, Paul V. Abbott

**Affiliations:** a *Neuroscience Research Center, Kerman University of Medical Sciences, Kerman, Iran; *; b *School of Dentistry, University of Western Australia, Perth, Australia*

**Keywords:** Anesthesia, Inferior Alveolar Nerve, Irreversible Pulpits, Pain, Root Canal, Succe

## Abstract

**Introduction**: Achieving successful anesthesia and performing pain-free root canal treatment are important aims in dentistry. This is not always achievable and therefore, practitioners are constantly seeking newer techniques, equipments, and anesthetic solutions for this very purpose. The aim of this review is to introduce strategies to achieve profound anesthesia particularly in difficult cases.** Materials and Methods:** A review of the literature was performed by electronic and hand searching methods for anesthetic agents, techniques, and equipment. The highest level of evidence based investigations with rigorous methods and materials were selected for discussion.** Results:** Numerous studies investigated to pain management during root canal treatment; however, there is still no single technique that will predictably provide profound pulp anesthesia. One of the most challenging issues in endodontic practice is achieving a profound anesthesia for teeth with irreversible pulpitis especially in mandibular posterior region. **Conclusion:** According to most investigations, achieving a successful anesthesia is not always possible with a single technique and practitioners should be aware of all possible alternatives for profound anesthesia.

## Introduction

There is no doubt that the most widely used method for pain management during dental, and particularly endodontic, procedures is to administer intraoral local anesthesia [1]. There are three general mechanisms for managing dental pain based on the pharmacological approach:

*i.* Blocking nociceptive impulses along the peripheral nerves.

*ii.* Reducing nociceptive input from the site of injury.

*iii.* Preventing pain perception in the central nervous system and reducing nociceptive input.

Pain management strategies during root canal treatment can be based on one or a combination of these mechanisms. *Blocking* the nociceptive impulses during root canal treatment is performed with the administration of local anesthesia [2], whereas *reducing* the nociceptive input is managed by prescribing a medicament such as a non-steroidal anti-inflammatory drugs (NSAIDs) to prevent prostaglandin formation at the site of injury [3, 4].

Pain management during and after root canal treatment is a major challenge for dental practitioners [5, 6]. Numerous high level of evidence investigations have been performed to overcome pain during and following root canal treatment [7]. Sometimes an intervention might be performed during treatment to manage post-operative pain for example, to prevent pain perception in the central nervous system which can be achieved by both prescribing a NSAID and by using a long acting anesthetic agent [3, 8].

Several anesthetic solutions are available on the market such as lidocaine with different concentrations of epinephrine, prilocaine, mepivacine, bupivacaine, articaine, and ropivacaine. The US Food and Drug Administration (FDA) approved articaine with epinephrine 1:100000 and 1:200000 in 2000 and 2006, respectively [9]. The choice of local anesthetics by dental practitioners is mostly based on the required duration of anesthesia, bone penetration, systemic conditions of the patient, and the presence and type of vasoconstrictor in anesthetic solution.

Several strategies have been introduced to provide profound anesthesia in order to enable root canal treatment to be performed as comfortable as possible. Numerous investigations have assessed various factors that affect the success of anesthesia and provide a more comfortable procedure, including:

1-Reducing the pain of the injection

   -Pain perception during administration of an anesthetic solution

   -Type of anesthetic solution

   -Size of the needle

   -Speed of injection

   -Topical anesthesia

2-Increasing the success rate of anesthesia

   -Intra-operative pain prevalence

   -Concentration of the epinephrine and volume of anesthetic solution

   -Premedication

   -Effect of the type of anesthetic solution on success of anesthesia

*A*) Maxillary arch

*B*) Mandibular arch

   -Effect of a combination of anesthetic agents and other medications for increasing the success rate of anesthesia

   -Supplementary anesthesia

*A*) Intraosseous techniques

*a.* Periodontal ligament (PL) injection

*b.* Intraosseous (IO) injection

*B*) Buccal infiltration

*C*) Intra-pulp injection

   -Anesthesia for different teeth

   -Other important factors

   -Clinical comments for proving profound anesthesia

The purpose of this literature review is to provide information regarding factors that may have influence on pain perception of the patients as well as how a clinician can provide profound anesthesia and manage pain during root canal treatment by using electronic and hand searching methods for anesthetic agents, techniques, and equipment. In most instances the highest level of evidence based investigations with rigorous methods and materials were selected for discussion.

## Reducing the pain of the injection

***Pain perception during administration of an anesthetic solution***

There are several factors that may influence pain perception during the injection of anesthetic solution including the type of anesthetic solution, the size of the needle, the speed of injection, and using topical anesthesia.

***The type of anesthetic solution***

Two percent lidocaine with 1:100000 epinephrine is one of the most popular anesthetic agents used in dentistry. Most dentists prefer to use anesthetic agents combined with a vasoconstrictor [10]. There is a possibility that the patients feel more pain when receiving certain types of anesthetic agents. Local anesthetics have different pH values and it is thought that lower pH values might cause a burning sensation during injection due to the acidic nature of the solution. Several investigations have confirmed differences in pain perception when different anesthetic solutions were injected [11-15]. In contrast, two other investigations have reported no significant difference in pain perception when different anesthetic solutions at different intraoral sites were used [16, 17]. One major shortcoming of some of these investigations is that they did not separate the pain felt during maxillary infiltration from that with inferior alveolar nerve block (IANB) injection. Because the site of injection may have an impact on pain during injection, using different anesthetic solutions at different sites and combining the results may not completely illustrate the pain associated with injection. In addition, these studies were not randomized double-blinded trials which may result in bias [13, 16, 17]. Only three investigations were randomized double-blinded clinical trials that reported a significant difference in injection pain when different anesthetic agents were used [11, 12, 15]. In these studies, prilocaine, articaine and plain lidocaine were associated with lower injection pain compared to 2% lidocaine with either 1:80000 or 1:100000 epinephrine.

The *site of injection* may be an important factor in injection pain. One study reported that maxillary buccal injections were associated with significantly less pain when plain 2% lidocaine was injected compared to 2% lidocaine with 1:80000 epinephrine. However, at the palatal site, no significance in pain of injection was reported with the same anesthetic agents [12]. In another study, no significant difference was reported between injection pain for maxillary buccal infiltration and IANB [11].

It can be concluded that when a site with less connective tissue (such as the palatal site in the maxilla) is injected, the type of anesthetic solution has no impact on injection pain. The studies with high levels of evidence reported significant differences in injection pain with different anesthetic agents.

***Size of the needle***

It has been reported that there is no significant difference in pain in adult subjects when 25 or 27 gauge (G) needles were used for IANB. In addition, there was no difference in pain when #25, #27, and #30 gauge needles were used for either buccal or palatal infiltrations of maxillary teeth [18]. However, in children having an IANB injection, a smaller sized needle (30 G) provided less unpleasant feeling and crying compared to a larger needle (27 G) whilst there was no significant difference with infiltration injections for maxillary molars [19].

It can be concluded that in adults needle size has no impact on injection pain; however, in children the site of injection may have some impact on the effect of needle size on pain during intraoral anesthesia injection.

***Speed of injection***

In the medical field, a higher speed of injection leads to an increase in the distribution of the drug. There has been a suggestion that a faster injection may expose a longer section of a nerve to the anesthetic solution and therefore there may be a higher rate success of local anesthesia [20]. Several randomized clinical trials reported that rapid injection have either significantly lower success rate [21] or no significant difference on IANB and incisive/mental nerve block success rate [22, 23]. However, the faster injections caused greater pain and discomfort during the injection [21, 23].

It can be concluded that the speed of injection has no significant effect on the success rate of anesthesia but faster injections provide greater pain and discomfort for patients.

***Topical anesthesia***

Numerous studies have been performed to evaluate the efficacy of topical anesthesia on injection pain [24-35]. These investigations have mostly focused on evaluating pain during needle insertion, during injection of the anesthetic solution, or both [24]. There is no general agreement regarding the efficacy of topical anesthesia to decrease the patient’s pain during needle insertion as well as injection. Results of some studies have been in favor of using topical anesthesia [25-30, 32, 34], whereas others have reported no significant influence on pain during either needle penetration or during injection of the anesthetic solution [25, 31-33].

Several factors might have influenced the efficacy of topical anesthesia including the time between application of topical anesthesia and the injection, the site of injection, and the type as well as the concentration of topical anesthetic agents [24, 25, 31, 32, 34].

Topical anesthesia should be placed over the mucosa at least 30 sec to 1 min before the injection [24]. Another important factor that may influence the feeling of pain during needle penetration and anesthetic solution injection is the site of the injection [24, 32, 34]. The degree of keratinization may have adverse effects on the efficacy of topical anesthesia. It seems that topical anesthesia for maxillary palatal injection, and for IANB injection has no positive effect on pain either during needle insertion or injection [24, 34]. The formulation of the topical anesthetic gel is an important factor that may influence the efficacy of the topical anesthesia [24, 25, 30, 35]. Several formulations such as 60% lidocaine or a combination of 2.5% lidocaine and 2.5% prilocaine have been reported to have greater efficacy compared to 20% benzocaine gel [25, 30].

In conclusion, despite topical anesthesia possibly being effective in only some sites of the oral cavity, its use is still recommended because it indicates to the patient that their dentist is trying to do everything possible to minimize pain during treatment. In addition, certain formulations of topical anesthesia such as a combination of 2.5% lidocaine and 2.5% prilocaine are more effective than conventional topical anesthetic agents.

***Increasing the success rate of anesthesia***

Most investigations on the success of anesthesia have used lip numbness as a sign of IANB success [4, 36-39]. These studies used either a crossover design or a randomized clinical trial. Crossover designed studies use different kinds of anesthetic agents or techniques in the same patients with sound teeth and healthy pulps at different times, whilst randomized clinical trials use different anesthetic agents or techniques in patients that have irreversible pulpitis with or without spontaneous pain in clinical treatment situations. After administration of anesthesia, cold tests or electric pulp testing (EPT) are used to evaluate anesthesia [4, 36-39]. In the crossover studies, no response to either the cold or electric pulp tests is assumed to be clinical success. In randomized clinical trials of teeth with irreversible pulpitis, no response to the cold or electric tests as well as no or minimal pain during access cavity preparation and root canal instrumentation has been used as the criteria for successful anesthesia. Most clinical trials have shown that both lip numbness and the lack of response to cold or electric tests are not good indicators of pulp anesthesia since most patients still had pain [4, 37-39]. One explanation might be that in teeth with irreversible pulpitis, the responses to EPT or cold tests are related to *fast* and *slow* silent Aδ-fibers, respectively [40]. Hence, it can be hypothesized that since the tetrodotoxin-resistant (TTX-resistant) sodium channels mostly appear on deeper nociceptive C-fibers, neither negative nor positive responses to EPT and cold tests indicate the success of anesthesia following administration of anesthetic agents [38].

***Prevalence of intra-operative pain***

Several studies have reported the prevalence of pain during root canal treatment [41-44]. Since even slight discomfort during treatment may be reported as pain, the prevalence of pain during treatment may not completely illustrate the quality of discomfort felt by the patients. Several studies have categorized pain during treatment in a manner that may indicate the real quality of discomfort during treatment. The prevalence of moderate to severe pain during root canal treatment has been reported to range from 11% to 35% [41-44]. It is important to note that the prevalence and severity of pain during treatment were generally rated in comparison to the levels of pre-treatment pain [43]. In one study, all patients reported pain during treatment [45], whereas Watkins *et al*. reported a pain prevalence of 22% [41]. The difference between reported pains in these two investigations was due to the anticipated pain during root canal treatment, and the use of various criteria for reporting pain. Most investigations have used the criterion that no or mild pain during the treatment indicates successful anesthesia [4, 37, 46-50], whereas some studies have reported all degrees of pain during treatment, no matter how mild or severe [45]. Two investigations have reported 12% and 35% moderate to severe pain during root canal treatment [42, 44]. Segura-Egea and associates reported that root canal treatment in teeth with irreversible pulpitis and acute apical periodontitis was significantly more painful than when treating teeth with necrotic and infected pulps [42]. In addition the duration of visit might significantly increase the risk of feeling pain during the treatment [42, 51].

Another cross-sectional clinical study reported significantly higher pain following step-back instrumentation when compared to rotary instrumentation [44]. In addition, treatment of molar teeth and teeth with irreversible pulpitis resulted in more intra-operative pain compared to treatment of single-rooted teeth and teeth with necrotic and infected pulps. However, the results of that study should be interpreted with caution because there was a small sample size in each group of the patients with different pulp and periapical conditions. Moreover, in that study, different anesthetic solutions were used with or without vasoconstrictor and these factors may provide shorter duration of anesthesia which may influence the results.

The effects of patients’ *age* and *gender*, and also the *dental arch* on the amount of pain during root canal treatment have been studied and the results have been conflicting. However, it is important to note that these factors may be associated with a higher risk of pain during treatment [41, 42, 44, 51].

In conclusion, patients with irreversible pulpitis and tenderness to percussion are most prone to feeling pain during root canal treatment.

## Concentration of the epinephrine and volume of anesthetic agents

Several investigations evaluated the efficacy of different concentration of epinephrine and volume of anesthetic agents [52-60].

***Concentration of the epinephrine:*** Using different concentrations of epinephrine has only been evaluated to a limited extent. Two studies that evaluated different concentrations of epinephrine for IANB and infiltration injections with either lidocaine or articaine have reported no significant differences between anesthetic agents with different concentrations of epinephrine [52, 53]. Both studies were cross-over studies but the teeth were asymptomatic and did not need root canal treatment.

Therefore, further research on teeth with irreversible pulpitis and in need of root canal treatment is needed to evaluate the effect of different concentrations of epinephrine on pulp anesthesia.

***Volume of anesthetic solution*** **: **There is no general agreement regarding the influence of the volume of anesthetic solution on the success rate of anesthesia. Several investigations have shown that higher volumes of anesthetic solution may increase the success rate [54-56], but others have reported no significant difference with higher volumes [57-60].

The type of anesthetic solution; may influence the results of the investigations. For instance, a recent investigation showed that increasing the volume of 4% articaine significantly improved the anesthesia success rate [55], whereas other studies did not report a significant difference when the volume of 2% lidocaine was increased [58, 60].

In the maxilla, the volume of anesthetic solution is very important for achieving longer duration and faster onset of anesthesia [58, 60].

Although several studies have been performed on the effects of increasing the volume of anesthetic agents with IANB injections, the heterogenicity of the anesthetic agents as well as the design of these studies and the site of injection prevents readers from obtaining a definite result. For instance, most investigations had cross-over designs that used intact teeth with healthy pulps [54, 58-60]. Only one study evaluated the effect of volume of anesthetic agent on teeth with irreversible pulpitis [56]. It has been shown that obtaining anesthesia in teeth with irreversible pulpitis is much more difficult compared to intact teeth with healthy pulps [39]. In addition, as mentioned above, EPT or cold tests used to evaluate anesthesia during crossover investigations are not accurate indicators of the success of anesthesia [4, 33, 37-39].

From the clinical standpoint, increasing the volume of the anesthetic solution may help to assure the patient that the dentist is doing everything in his/her power to make the treatment as painless as possible. More high level evidence studies on teeth with irreversible pulpitis are needed to determine whether the volume of anesthetic agent has any effect on the success rate of anesthesia.

## Premedication

Several types of medication have been used to increase anesthesia success including using benzodiazepines (triazolam, alprazolam and diazepam), NSAIDs, and corticosteroids [4, 36, 61-71].

The concept of using benzodiazepines is based on reports that have shown anxiety may decrease the success of anesthesia and using a medication to overcome this anxiety may increase anesthesia success [20, 61, 72, 73]. Despite the superiority of triazolam compared to diazepam and placebo in decreasing patients’ anxiety, none of the previous studies reported any significant effects on IANB anesthesia following premedication with benzodiazepines [67, 69].

The concept of using NSAIDs and corticosteroids as premedications to improve success of anesthesia seems rational because the amount of prostaglandins is significantly increased in inflamed pulps compared with normal healthy pulps. It has been confirmed that higher levels of prostaglandins can affect TTX-resistant receptors and decrease nerve responses to anesthetic agents [39, 74]. Therefore, any medication that can affect the amount of prostaglandins may increase the success rate of anesthesia [75]. Several studies have confirmed that NSAIDs such as ibuprofen have anti-inflammatory effects. However, there is no general agreement regarding the efficacy of premedication on the success of anesthesia. Some studies report positive influences of NSAIDs medication [4, 36, 65, 66], whereas others report no significant difference between placebo and NSAIDs on the success rate of anesthesia [62-64, 68, 70, 71]. The difference between the inclusion criteria and the type of NSAIDs used might be the reason for conflicting results [4, 65, 76]. Parirokh *et al*. suggested that premedication for patients with irreversible pulpitis but with no spontaneous pain may have more beneficial effect compared to those patients who have spontaneous pain, because premedication with NSAIDs would have no positive effect on previously formed TTX-resistant sodium channels [4]. Results of another study showed no significant difference when ibuprofen was used as premedication for patients with no spontaneous pain but the level of significance (*P*=0.055) in the ibuprofen group was very close to be significant compared to the placebo group [70].

The type of medication may also affect the results of studies that have evaluated the efficacy of premedication with NSAIDs on anesthesia success [65, 76]. A meta-analysis on the effect of premedication with NSAIDs on the success of IANB showed dosages of 600 to 800 mg of ibuprofen, 75 mg indomethacin, 8 mg lornoxicam, and 50 mg of diclofenac potassium significantly increase the success rate of IANB, whereas other NSAIDs such as ketorolac, a combination of ibuprofen and acetaminophen, as well as acetaminophen alone had no significant effect on the success of anesthesia compared to placebo [76]. It should be noted that the meta-analysis only included seven studies that had been performed until July 2011 and since that time, two new studies have been performed which reported no significant effect of NSAIDs on IANB success rate [70, 71].

Only one study has investigated the use of corticosteroid as premedication prior to anesthesia with an IANB injection [70]. Despite a significantly higher success rate than placebo, all patients were not completely anesthetized when dexamethasone was used as the premedication. Dental practitioners should always consider the risks and the benefits of drug administration, especially for corticosteroids [39].

Therefore, based on high level of evidence studies, pretreatment with some types of NSAIDs may have a positive influence on the success of anesthesia when treating irreversible pulpitis provided that the patient has no spontaneous pain.

**Effect of the type of anesthetic solutions on success of anesthesia**

It is generally accepted that teeth with irreversible pulpitis are the most challenging ones to be anesthetized, whereas crossover design studies have typically used healthy pulps to evaluate the efficacy of anesthesia [39, 77]. Therefore, in this review the results of these investigations have been addressed separately. In addition, mandibular teeth are more difficult to anesthetize than maxillary teeth, and for that reason they have also been addressed separately [77].

**Maxillary arch**

***Crossover designed studies of teeth with healthy pulps:*** No significant difference in the success rate of anesthesia in maxillary canine teeth has been reported when comparing 4% articaine with 4% prilocaine (both with 1:200000 epinephrine) for buccal infiltration injections [78]. When 4% articaine was compared with 2% lidocaine (both with 1:100000 epinephrine) in the maxillary arch, articaine showed a significantly higher success rate for lateral incisors, but there was no significant difference for the first molar teeth [79]. An investigation that compared 2% lidocaine with either 1:50000 or 1: 80000 epinephrine and 3% mepivacaine, reported no significant difference between the anesthetic agents for maxillary lateral incisors and first molars [80]. A comparison between 2% lidocaine with 1:100000 epinephrine and 3% mepivacaine when administered as a maxillary second division nerve block with high tuberosity approach, resulted in no significant difference between the efficacy of the anesthetic solution for maxillary molars and premolars [81]. When 0.5% bupivacaine with 1:200000 epinephrine was compared with 2% lidocaine with 1:100000 epinephrine, bupivacaine showed significantly lower success rates for lateral incisors but no significant difference for maxillary first molars [82]. No significant difference between the success rates of anesthesia for maxillary incisors and canines was reported when 0.5% plain ropivacaine was compared to 4% articaine with 1:100000 epinephrine [83].

In conclusion, most studies have reported no significant difference between different anesthetic agents for the success rate of anesthesia in maxillary teeth when testing healthy pulps, although two studies [79, 82] reported that different types of teeth may respond differently to the various anesthetic formulations with the same infiltration injection technique.

***Studies of teeth with irreversible pulpitis*** **: **Only three studies have been performed on maxillary teeth with irreversible pulpitis. Two studies reported no significant differences between 4% articaine with 1:100000 epinephrine and either 2% lidocaine with 1:80000 [12] or 2% lidocaine with 1:100000 epinephrine when treating maxillary anterior, premolar and molar teeth [84]. In contrast, another study reported a significantly higher success rate for 4% articaine with 1:100000 epinephrine when treating maxillary molars and premolars [85].

The limited number of studies with high level of evidence does not allow any firm conclusions to be drawn regarding anesthesia techniques for maxillary teeth with irreversible pulpitis. More high level evidence based investigations need to be performed.

## Mandibular arch

***Crossover designed studies of teeth with healthy pulps:*** Variable results have been reported in several studies. No significant difference was found on anesthetic efficacy between 4% prilocaine, 3% mepivacaine and 2% lidocaine with 1:100000 epinephrine for teeth with healthy pulps following IANB injections [86]. No significant difference on the success rate of IANB anesthesia was reported when 3% mepivacaine was compared with 2% lidocaine with either 1:80000 or 1:100000 epinephrine [87, 88]. The use of 4% articaine for incisive/mental nerve blocks provided significantly higher success than 2% lidocaine (both solutions with 1:100000 epinephrine) for mandibular lateral incisors, canines and both first and second premolars [89]. Meanwhile, no significant difference was reported for mandibular canine teeth when 4% articaine was compared with 4% prilocaine both with 1:200000 epinephrine for infiltration injection on the buccal of the teeth in both mandible and maxilla [78]. No significant difference was reported in the success rate for mandibular first molar anesthesia following IANB injections with 2% lidocaine with 1:80000 epinephrine compared to 4% articaine with 1:100000 epinephrine following either buccal, or buccal and lingual infiltrations [90].

In conclusion, most crossover design studies have reported no significant differences for the efficacy of different anesthetic agents when used for IANB injections.

***Studies of teeth with irreversible pulpitis:*** Several studies have reported no significant difference on the success rate of either Gow-Gates block [84] or IANB anesthesia [91, 92] when 4% articaine with 1:100000 and 2% lidocaine with 1:100000 have been compared. Similarly, no significant differences have been reported between 0.5% bupivacaine versus ethidocaine, both with 1:200000 epinephrine [93], and 0.5% bupivacaine with 1:200000 epinephrine versus 2% lidocaine with 1:100000 epinephrine [38].

In conclusion, most studies have reported no significant difference when different anesthetic agents were used for achieving IANB anesthesia as primary injection when treating mandibular teeth with irreversible pulpitis.

A meta-analysis compared articaine and lidocaine and reported higher success rate of anesthesia for the former anesthetic formulation when used in infiltration injection, whereas for inferior alveolar nerve block articaine was only superior to the lidocaine in asymptomatic or normal pulp teeth [94].

## Effect of a combination of anesthetic agents and other medications for increasing the success rate of anesthesia

Addition of anti-inflammatory drugs might rationally improve the success of anesthesia because they tend to prevent formation of prostaglandin as a very important mediator to affect formation of TTX-resistant sodium channels. However, from the clinical standpoint, this is not always useful. As an example, it has been reported that the addition of dexamethasone did not improve anesthesia success for mandibular molar teeth with irreversible pulpitis [47]. In addition, even when a NSAID is used for its anti-inflammatory effect, some adverse side effects such as severe pain during injection may occur [95]. A study has been performed to determine whether the success of IANB anesthesia for irreversible pulpitis in mandibular molar teeth could be increased by using a supplementary buccal injection of dexamethasone, 4% articaine with 1:100000 epinephrine alone, or 4% articaine with 1:100000 epinephrine combined with ketorolac. Significant higher success rates of anesthesia were obtained when either articaine alone or in combination of with ketorolac was used [47].

Another reason for using additives in anesthetic solutions is to decrease the pain of the injection and increase the speed of onset of anesthesia. Some anesthetic agents (for example, lidocaine) have an acidic pH and some researchers believe that acidic pH increases the pain of injection and slows the onset of anesthesia [39]. A crossover investigation on the addition of sodium bicarbonate to buffer 2% lidocaine with 1:100000 epinephrine showed no significant improvement for pain and onset associated with maxillary buccal infiltrations for canine teeth [96].

The route and method of administration of additives may be important to increase their efficacy for example, the addition of hyaluronidase to lidocaine provided no significant improvement in anesthesia success and induced negative side effects such as trismus and post-operative pain [97]. However, when the hyaluronidase was injected shortly after injection of the anesthetic solution, the duration of anesthesia improved [98, 99].

The addition of meperidine did not significantly improve the success of IANB anesthesia for mandibular molars and premolars with irreversible pulpitis [100].

The addition of mannitol to anesthetic agents was tested because of its ability to temporarily dissolve the perineural membrane and thereby increase the penetration of the anesthetic solution [39]. When mannitol was added to lidocaine, there was an increase in success rate of anesthesia in mandibular molars and premolars with both healthy pulps and irreversible pulpitis [49, 101].

Some researchers have added diphenhydramine to enhance the anesthetic effect via action on the sodium channels. However, this combination was not as effective as lidocaine alone and there were also side effects such as injection pain as well as post-treatment pain and discomfort [102].

In conclusion, some additives have a positive effect on anesthesia success and duration but more studies of their possible benefits and risks are needed.

## Supplementary anesthesia

Local anesthesia success is an important part of daily practice for every dentist. Most investigations on anesthesia success in dentistry have reported various percentages of failure when different types of anesthetic techniques and agents were used. Kaufman and associates reported 13% of general practitioners encountered failure of anesthesia during five days per week of practice and, most importantly, 10% of the dental procedures could not be continued because of these anesthesia failures [103]. The most common failures occurred with IANB injections.

Sometimes, particularly in teeth with spontaneous pain and irreversible pulpitis, routine anesthetic techniques cannot provide enough pain relief for the patients when the access cavity is being prepared or during root canal instrumentation [4, 37, 38, 104]. Hence, in order to try and overcome these failures, supplementary anesthetic techniques have been introduced [2].

**Intraosseous injection techniques**

Two forms of intraosseous injection techniques have been described including the periodontal ligament (PDL) injection and the intraosseous (IO) injection. The latter technique usually involves the use of specific devices such as the Stabident, X-Tip, and Intraflow devices [2, 105].

***Periodontal ligament (PDL) injection:*** The periodontal ligament injection, also known as the *intra-ligamentary* injection technique [2, 73], is actually an intraosseous injection during which the anesthetic solution is injected via periodontal ligament. The solution reaches the pulp nerves through the natural cribriform plates of the tooth socket wall to the cancellous bone [2]. Results of a web-based survey showed that the PDL injection is the most popular supplementary technique used by members of the American Association of Endodontists (AAE) in the USA [105]. Several investigations have been performed on the efficacy of the PDL injection [73, 106-111]. Anesthesia after PDL injection is usually achieved after 30 sec [112, 113]. The most important points regarding this technique are the positioning of the needle and to inject the anesthetic agent with force. The clinician should feel resistance to injection during the procedure and considerable force should be required to deposit the solution [112]. If the needle is incorrectly placed and no pressure is felt when injecting, then the solution will not be forced into the bone and will not reach the pulp nerves.

The use of a PDL injection in addition to an IANB injection significantly increased the success rate of anesthesia during the first 23 min following the injection [108]. Placement of a combination of lidocaine and prilocaine cream at the PDL injection site, when no other anesthetic technique is used, resulted in significantly lower injection pain compared to an ointment of 5% lidocaine [73]. No significant difference was reported between either injection pain or post injection pain in the mandibular first molar after using either 4% articaine or 2% lidocaine (both with 1:100000 epinephrine) for the PDL injection [109]. Two studies have reported between 56 to 70% of mandibular posterior teeth with irreversible pulpitis that still had pain following conventional anesthetic techniques were successfully anesthetized with the PDL injection [110, 111].

In conclusion, the PDL injection is a popular supplementary technique that increases the success rate of anesthesia, although it does not always result in profound anesthesia.

***Intra-osseous (IO) injection: ***The IO injection is one of the most successful methods among all supplementary anesthesia techniques used to overcome continued pain following conventional anesthetic techniques in dentistry [2, 111, 114-117].

Several systems have been introduced for IO injections including Stabident (Fairfax Dental Inc., Miami, FL), X-Tip (Dentsply International Inc, Tulsa, OK, USA), IntraFlow (Pro-Dex Inc, Santa Ana, CA), and Quick Sleeper 2 (DHT, Cholet, France) [111, 116-120] devices. Most studies of the IO technique have used it as a supplementary anesthetic technique but a few studies have successfully used IO as the primary anesthesia technique [119-121]. 

Repeated IO injections significantly improved pulp anesthesia in one study [121]. However, the major drawback of the two studies that used the IO injection as the primary anesthetic technique was that they anesthetized and tested teeth that did not require root canal treatment as they had healthy pulps [119, 121]. Another investigation that used the IO injection as a primary technique was a cohort investigation that used the technique for teeth in need of restoration, endodontic treatment, or extraction in children and adolescents [120]. It is well known that providing anesthesia for endodontic treatment is the most challenging problem compared to the other dental procedures such as restoration and even tooth extraction [2]. It has also been shown that the efficacy of IO injections on the success rate of pulp anesthesia is not the same in different parts of the oral cavity owing to differences in the cancellous bone spaces [2].

The IO injection has systemic side effects such as heart rate elevation [111, 118, 122]. In addition, the IO injection is not easy to perform and mostly needs special equipment. Other drawbacks for the IO injection are pain and discomfort after injection and a potential to damage the teeth during perforation of bone by the perforator [2]. It is also difficult to use when rubber dam is in place in situations such as when pain is encountered during access cavity preparation or during instrumentation of the root canals.

In conclusion, although the IO injection provided the highest rate of anesthesia as a supplementary injection, it needs special equipment, is difficult to perform and may induce post-operative pain and discomfort.

## Buccal infiltration

Buccal infiltration has been used either as a primary technique [31, 46, 90, 123-127] or supplementary anesthesia [-] for anesthetizing mandibular molar teeth. Two studies also have tested both IANB and buccal infiltration [37, 133]. Most studies report that using 4% articaine as supplementary anesthesia, significantly provided higher success rates compared to 2% lidocaine [129, 132, 134]. However, in contrast, another study found no significant difference between lidocaine and articaine for buccal infiltrations [128].There was no significant difference between articaine as primary buccal infiltration anesthesia compared with lidocaine for IANB anesthesia [46, 90].

In conclusion, when a practitioner decides to use an infiltration injection for supplementary anesthesia for mandibular molars, articaine may provide better results compared to 2% lidocaine.

## Intra-pulpal injection

The intra-pulp injection (IP) technique should be considered as the last resort for pulp anesthesia and should only be used when all other supplementary techniques have been tried but without success.

The most important point for this technique to be successful is to inject the solution into the pulp with* force*. If pressure or resistance to injection is not felt by the clinician, then the solution is generally not reaching the pulp and is likely to be flowing out of the pulp chamber and back into the access cavity. It has been confirmed that it is not necessary to inject an anesthetic agent inside the root canal because even an injection of saline can provide anesthesia provided it is injected with force [135, 136].

As mentioned previously, the IP injection is very painful and therefore should be performed as the last resort during endodontic treatment.

Another disadvantage of IP anesthesia is its short duration of action [2, 79]. Therefore, following the use of an IP injection, it is essential to remove the pulp from all the root canals as quickly as possible to prevent the need to repeat the IP injection. Repeated injections are less likely to work as the opening into the pulp and each canal becomes larger since there is less chance of creating pressure during the injection.

An investigation on dogs’ teeth reported that following IP injection, 62% of the anesthetic agent reached the apex and therefore it is possible to extrude or pulp remnants or debris into the periapical tissues [137]. Therefore, the IP injection is not recommended in teeth with *partial pulp necrosis* if the patient feels pain during root canal negotiation or instrumentation. Some authors have recommended using topical anesthesia inside the root canal instead of injecting an anesthetic solution with force [138, 139]. DeNueizo suggested the insertion of topical anesthesia into the root canal space as a clinical adjunct during root canal preparation to provide pain relief if the patient did not respond well to all other supplementary anesthetic techniques [139].

However, there are several drawbacks of the technique, including the possibility of extrusion of the topical gel into the periapical tissues and interference with the adherence of the root filling to the canal walls.

## Anesthesia techniques for different teeth

Various specific techniques have been described to help achieve predictable anesthesia in different teeth. For mandibular central and lateral incisors, a combination of buccal and lingual infiltrations provides significantly higher rates of success of anesthesia compared to either a labial or a lingual infiltration [140, 141].

The *palatal-anterior superior alveolar (P-ASA)* injection has been described for anesthetizing maxillary incisors and canines. However, this technique has a potential for being painful during needle insertion, and also during and after injection. Moreover it can cause swelling, numbness, and parasthesia of the incisive papilla even when a computer-aided injection system was employed [142].

For maxillary molar teeth, the combination of buccal and palatal injections significantly increased the duration of anesthesia from 21 to 57 min [143]. *Greater palatine *and* high tuberosity second division nerve blocks* are effective techniques for anesthetizing the first and second maxillary molars in most cases, whereas only about two-thirds of second premolars were anesthetized with these techniques. No significant difference was found between the efficacy of these techniques, although more post-injection pain was reported with the high tuberosity second division injection technique [144].

Higher success rate of anesthesia for mandibular molar has been reported when the *Gow-Gates mandibular nerve block* technique was compared with either conventional IANB or buccal and lingual infiltrations [125]. However, two other studies found no significant differences between different anesthetic techniques for mandibular molars [145, 146].

No significant difference was found between the *posterior superior alveolar nerve block*, *buccal infiltration* and *buccal plus palatal injection* for anesthetizing maxillary first molars with irreversible pulpitis [147].

One study used frequency dependent stimulation for blocking inferior alveolar nerve following IANB and found no significant increase in pulp anesthesia in mandibular teeth [148].

In conclusion, dentists should employ techniques that provide higher success rates while having less injection pain and less post-injection pain and discomfort for the patient. Supplementary or alternative techniques should be used when the first injected is not successful in providing profound anesthesia.

## Other important factors

There is an old belief that soft tissue massage following administration of anesthesia may have some beneficial effect on the speed of onset of anesthesia and its success rate. However, the results of a recent randomized clinical trial showed no significant difference in the speed of onset, success and discomfort following anesthesia using soft tissue massage following an incisive/mental nerve block [149].

A decline or decrease in pulp anesthesia (non-continuous anesthesia) has been reported in several studies with cross-over designs where patients initially reported no pain to an EPT evaluation but they sometimes reported later pain with the same test [39, 88, 134, 150]. This is similar to the clinical scenario where patients sometimes report pain in the middle of a treatment visit. In order to overcome this problem, three studies tested repeated injections of the anesthetic solution 20 to 30 min after the first injection. All three studies reported significant improvement in pulp anesthesia for maxillary lateral incisors, mandibular premolars, and first molars following infiltration injections [150-152].

Finally, the administration of 30%-50% nitrous oxide has been reported to significantly increase the success rate of IANB injections [50].

## Clinical comments to help provide profound anesthesia

A dental practitioner should always be up to date to employ all possible data to manage pain during and after root canal treatment [2, 8, 39, 153]. There are several clinical comments for pain management during root canal treatment that are useful to be remembered:

- If the patients observe that the dentist and dental staff all doing their maximum efforts for their comfort, their co-operation will increase and this will help the practitioner to use alternative routes to overcome the pain during treatment. The practitioner may also have some benefits from the placebo effect of their behavior [154].
- Always be honest to your patients regarding the possibility of evoking pain during root canal treatment. If the practitioner is going to use an IP injection as the last resort and he thinks the injection might be painful, then the patient should be informed of what to expect in advance of the injection.
- It is important to ensure the patient does not have pain when he/she is dismissed from the office. If necessary, administer another injection at the end of the treatment visit to ensure immediate post-operative pain relief [8].
- The speed of injection has no significant difference on anesthesia success. However, a slow injection is significantly more comfortable for the patient than a rapid one [22].
- Pain during injection has no significant effect on the success rate of anesthesia [33]. Therefore, the patient should be assured that if the anesthetic injection induced some pain, it has nothing to do with the success of anesthesia.
- There is no significant difference on the efficacy of anesthesia between a needle bevel directed away from the ramus or a bi-directional needle rotation technique during IANB injection [155].
- When infiltration injection is used, the length of the tooth that is going to be anesthetized should be considered. There is a strong possibility of anesthetic failure if the needle is placed shorter than the estimated length of the tooth.
- If a patient has a history of difficulty in obtaining anesthesia, supplementary techniques should be considered in advance in order to help avoid encountering anesthetic failures [20].
- If the practitioner assumes that the root canal treatment may take longer than expected and is time consuming, whenever possible, the plain form of the anesthetic agents should be avoided because they provide short duration of anesthesia [39, 81].

## Conclusion

Providing profound anesthesia is the ultimate goal of every dental practitioner and most desirable for patients. In order to achieve this goal, the whole dental team and the patient should work together act as a team to prevent failure and provide profound anesthesia. The practitioner should be aware of the different routes and injection techniques for increasing the depth of anesthesia as well as be confident in managing the patient’s anxiety so the dental treatment can be performed as painlessly as possible.
